# The Geography of Primary Hepatic Neoplasms Treatments in Canada: Changes in Latitudes and Changes in Attitudes

**DOI:** 10.1155/2017/9365657

**Published:** 2017-07-26

**Authors:** Matthew Cwinn, Gordon Walsh, Sheikh Hasibur Rahman, Michele Molinari

**Affiliations:** ^1^Department of Surgery, Dalhousie University, Halifax, NS, Canada; ^2^Surveillance and Epidemiology Unit, Cancer Care Nova Scotia, Halifax, NS, Canada; ^3^Department of Surgery, University of Pittsburgh, Pittsburgh, PA, USA

## Abstract

**Background:**

Studies on treatment modalities for primary hepatic neoplasms (PHN) in Canada are lacking. Our primary aim was to analyze the age-standardized incidence of hepatic resection, ablation, transplantation, and embolization for PHN between 2002 and 2013. Secondary aim was to evaluate temporal trends for these treatment modalities.

**Study Design:**

National Canadian Cancer Registries were accessed for relevant epidemiological data. Age-standardized incidence of treatment ratios (SIRs) was calculated and comparisons were performed for Atlantic Canada, Ontario, the Prairies, and British Columbia.

**Results:**

British Columbia recorded the highest SIRs for ablation (1.9; 95% CI 1.8–2.0), hepatic resection (1.2; 95% CI 1.1–1.3), and transarterial locoregional therapies (2.8; 95% CI 2.4–3.2). For hepatic resection, the lowest SIR was found in Atlantic Canada (0.7; 95% CI 0.6–0.9), while the Prairies recorded the lowest estimate for transarterial therapies (0.2; 95% CI 0.1–0.4). Liver transplantation had the highest SIR in Ontario (1.5; 95% CI 1.3–1.6) and the lowest SIR in British Columbia. No significant temporal changes in SIRs were observed for any of the treatments except for transarterial therapies.

**Conclusions:**

Treatment of PHN in Canada differs by geography. Variations might be due to differences in expertise or access to therapeutic modalities.

## 1. Introduction

Hepatocellular carcinoma (HCC) and intrahepatic cholangiocarcinoma (ICC) represent the majority of primary hepatic neoplasms (PHNs) [1]. In North America, HCC affects 85% of patients with PHN, while ICC accounts only for 15% of cases [2]. Worldwide, HCC represents one of the most frequent causes of cancer-related deaths in low-income countries [3]. Its incidence is the highest in Africa and Asia [4] and increasing in most Western nations [5]. Overall, the prognosis of HCC and ICC depends on stage of diagnosis and geographical area in which the patient lives. Five-year survival for patients with HCC ranges from less than 5% in developing countries [6, 7] to 47–53% for patients who undergo surgical resection [8] and up to 84% for liver transplant recipients. In contrast to most of other solid tumors, PHN can be treated with a variety of interventions [9]. These include transplantation, hepatectomy, and ablation [10–14]. Several therapies, such as transarterial catheter embolization (TACE) [15, 16], radioembolization [17, 18], stereotactic external beam radiation [19], ablation [20–22], and systemic chemotherapy [23, 24], are used as palliative measures or, in selected cases, as a bridge to liver transplantation [9, 15, 16, 25].

Management of patients with PHN is complex [13] as the majority have cirrhosis or some degree of liver dysfunction that can rapidly progress to liver failure when treated with cancer-directed therapies [26]. Because of this risk, most patients require tertiary or quaternary medical centers where coordinated multidisciplinary teams are available [27]. Several studies have shown that management of patients with PHN is variable and depends on access to health care, patients' socioeconomic factors, and availability of liver grafts [28–30]. Other barriers also play a role, and a considerable proportion of patients do not receive cancer-directed treatment [31], even when eligible [32]. Since the burden of PHN has significantly increased over the last decades [33], Canadian guidelines recommend screening high risk patients every 6 months with abdominal ultrasound and serum alpha fetoprotein levels [34]. Yet there is a lack of population-based studies on how patients are treated at a national level. Canada's health care system is publicly funded and provides coverage for all Canadian citizens, but funding is administered locally on a provincial or territorial basis [35]. Since economic indicators pertinent to different Canadian provinces vary, so do resources dedicated to health care [35, 36]. Theoretically, comparable health care systems and treatments should be available to all Canadian citizens, but this might not be the case because of differences in health care resources and expertise in different regions [5]. We hypothesized that due to the complexity of PHN management, heterogeneity in health care services might be associated with significant variations in treatment modalities. Therefore, the primary aim of this study was to analyze the age-standardized incidence of common PHN treatments during the time period between 2002 and 2013 in different geographical areas in Canada. The secondary aim was to evaluate temporal trends for common PHN treatment modalities.

## 2. Study Design

Data on modalities used to treat PHN in Canada were extracted from national databases, which were the Canadian Chronic Disease Infobase (CDIC) at the Public Health Agency of Canada (PHAC) and the Canadian Institute of Health Information's (CIHI) Discharge Abstract Database (DAD) and National Ambulatory Care Reporting System (NACRS). A detailed description of how pertinent data were obtained for the period between 2002 and 2013 has been shown in a previous publication by our group [37]. Briefly, CDIC CUBES (http://infobase.phac-aspc.gc.ca/cubes/index-eng.html) were investigated in addition to direct personal communications with data managers and statisticians at PHAC when data pertinent to primary and secondary outcomes were not available online.

Statistics on treatment modalities were obtained through the DAD and NACRS datasets for all patients admitted to a Canadian hospital with the diagnosis of primary liver tumor and treated with any of the following modalities: transplantation, hepatectomy, ablation, or pharmacologic therapy. Canadian Classifications of Health Interventions [38] codes were used for this study and are summarized in Table 1.

Patients who underwent liver transplantation were included if they received whole or partial organ grafts from either cadaveric or living donors. Hepatic resections included anatomical and nonanatomical hepatectomies performed either laparoscopically or by open surgery. Ablation procedures included all percutaneous, laparoscopic, or open interventions targeting primary liver tumors with the main goal of causing necrosis of neoplastic tissues by injecting chemicals (e.g., acetic acid or ethanol) or thermal injury (e.g., cryoablation, radiofrequency ablation, or, more recently, microwave technology). Pharmacologic therapies included the use of transcatheter hepatic artery infusions of embolizing agents with or without the addition of chemotherapeutic or radioactive particles. All interventions for the treatment of PHN during the study period were identified using the International Classification of Diseases and Related Health Problems (ICD) version 10 (ICD10) that was introduced in Canada in 2000. ICD-Oncology code C22.0 was used to identify patients with PHN that included the following diagnostic groups: hepatocellular cancer, hepatic cell carcinoma, mixed hepatocellular carcinoma, fibrolamellar carcinoma, hepatocholangiolitic carcinoma, mixed bile duct with hepatocellular carcinoma, cholangiocarcinoma with hepatocellular carcinoma, cholangiohepatoma, hepatocarcinoma, hepatocholangiocarcinoma, and malignant embryonal hepatoma.

Age-standardized incidence of treatment ratios (SIRs) were calculated, accounting for differences in the age structure of the populations in different geographical areas being compared during the time periods. Comparison of Canadian regions was performed by clustering provinces into four areas. The first was Atlantic Canada, which included the provinces of Nova Scotia, New Brunswick, Prince Edward Island, Newfoundland, and Labrador (estimated population 2,280,000). The second region was Ontario (estimated population 13,900,000) and the third region was the Prairies, which included the provinces of Manitoba, Saskatchewan, and Alberta (estimated population 6,720,000). The fourth region was represented by the province of British Columbia (estimated population 4,750,000). Data from Quebec were not made available; therefore, this province was excluded. Geographic clustering was requested by the Canadian agencies providing the data to protect patients' confidentiality, as some provinces had a small number of patients diagnosed with PHN during the study period. Also, this was necessary for statistical analysis, as it allowed the creation of larger size populations for comparisons.

To adjust for population characteristics by gender and age, SIRs were calculated to determine whether the number of procedures performed in a given year or within a particular region was significantly higher or lower than expected. A region's expected case count for each treatment modality for the time interval was calculated based on population characteristics using the midpoint of the study interval and corresponding countrywide average age-specific rates. The expected number was the overall rate for the country multiplied by the number of individuals living in the geographical area and interval time of interest. To control for sex and age, the calculations were performed separately for men and women and, within each gender, adjusted for age. SIR was calculated using the formula: SIR = (observed cases/expected cases) × 100 [39]. A SIR value of 1.0 indicated that the treatment modality in the region or time interval was equal to the expected countrywide average adjusted for age and gender. SIR values of more or less than 1.0 indicated that the treatment modality was above or below the expected countrywide average adjusted for age and gender. SIR and the approximate 95% lower and upper confidence limits were calculated by applying the Wilson and Hilferty approximation for chi-square percentiles [40] and obtained using an online program (Epi_Tools.XLSX) created by the Boston University School of Public Health in Excel (Microsoft®) platform [41]. Two-tailed significant statistical differences were identified when 95% confidence intervals of the SIR did not include the value of 1.0 (*P* value ≤ 0.05).

## 3. Results

During the study period, the mean estimated Canadian population was 33 million (SD 1.1 million), with age-standardized incidence of PHN equal to 4.8 per 100,000 individuals (7.3 for males and 2.3 for females per 100,000). A total of 9,980 patients were diagnosed with PHN and 4,338 treatments were recorded in the national cancer registry during the same period. Ablation was the most common treatment, performed 2,005 times (46.2% of treatments); hepatic resection was performed 1,642 times (37.9% of treatments); liver transplantation was performed 438 times (10.1% of treatments); and transarterial locoregional therapies were performed 253 times (5.8% of treatments).

**Figure 1:**
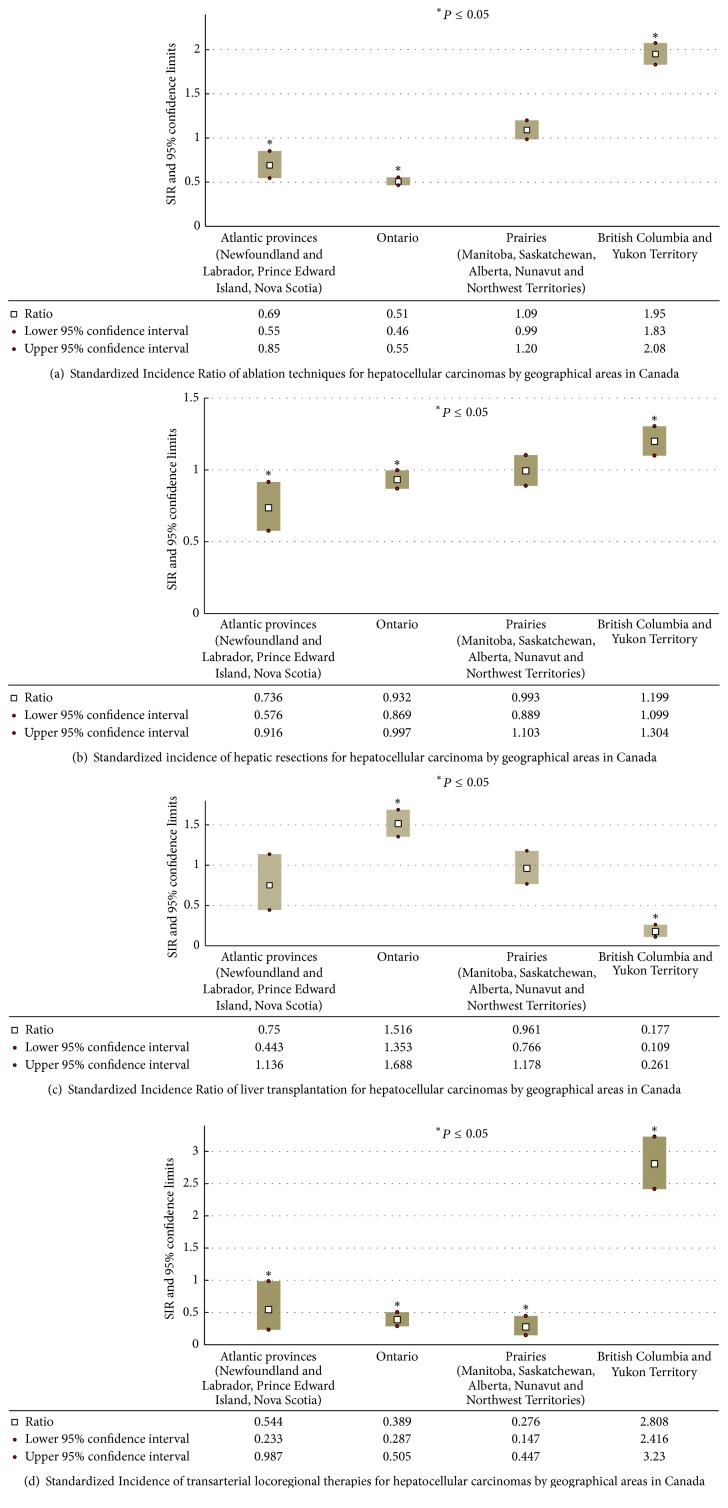
Standardized Incidence Ratio (SRI) and respective 95% confidence limits for ablation (a), hepatic resection (b), liver transplantation (c), and transarterial locoregional therapies (d) for the treatment of primary hepatic liver tumors in Canada during the period between 2002 and 2013. Values of SRI equal to 1.0 indicated that the treatment modality in the region was equal to the expected countrywide average adjusted for age and gender of the population. SIR values more or less than 1.0 indicated that the treatment modality was above or below the expected countrywide average adjusted for age and gender. Statistically significant differences were identified when 95% confidence intervals of SIR did not include the value of 1.0 (^*∗*^*P* value ≤ 0.05).

Across the four geographical areas, British Columbia recorded the highest value of SIRs for ablation (1.9; 95% CI 1.8–2.0), hepatic resection (1.2; 95% CI 1.1–1.3), and transarterial locoregional therapies (2.8; 95% CI 2.4–3.2) (Figures 1(a), 1(b), and 1(d)). For hepatic resection, the lowest SIR was found in Atlantic Canada (0.7; 95% CI 0.6–0.9), while the Prairies recorded the lowest estimate for transarterial locoregional therapies (0.2; 95% CI 0.1–0.4). Liver transplantation had the highest SIR in Ontario (1.5; 95% CI 1.3–1.6) and the lowest one in British Columbia (0.1; 95% CI 0.1–0.2) (Figure 1(c)).

**Figure 2:**
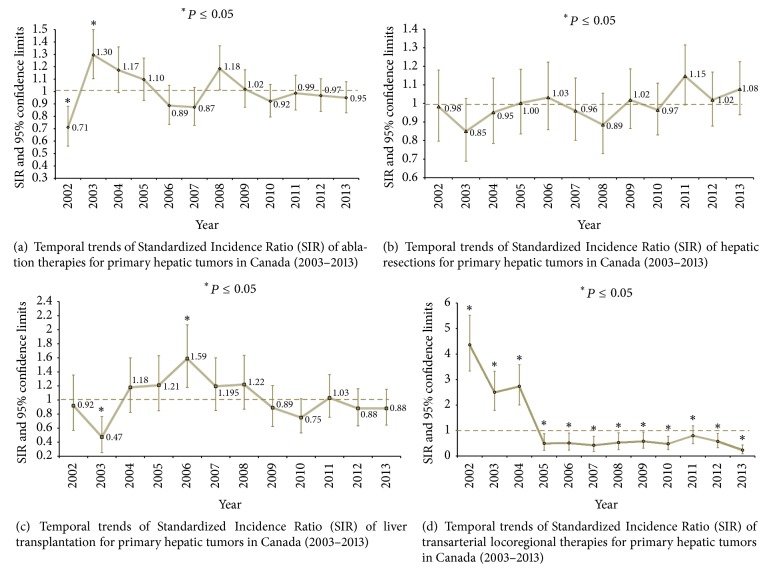
Temporal trends of the Standardized Incidence Ratio (SRI) and respective 95% confidence limits for treatment modalities of primary hepatic tumors in Canada over the period between 2002 and 2013. Over the study period, there were no significant temporal changes in SIRs for ablation, hepatic resection, or liver transplantation (a, b, c). On the other hand, transarterial locoregional therapies experienced a significant decline between the years 2004 and 2005 and national SIR remained below the expected value for all the following years (d). SRI values equal to 1.0 indicated that the treatment modality during the time period was equal to the expected countrywide average adjusted for population age and gender. SIR values more or less than 1.0 indicated that the treatment modality was above or below the expected countrywide average adjusted for age and gender. Statistically significant differences were identified when 95% confidence intervals of SIR did not include the value of 1.0 (^*∗*^*P* ≤ 0.05).

Over the study period, there were no significant temporal changes in SIRs for ablation or hepatic resection liver transplantation (Figures 2(a), 2(b), and 2(c)). On the other hand, transarterial locoregional therapies experienced a significant decline between the years 2004 and 2005 and the national SIR remained below the expected value for all the following years (Figure 2(d)).

## 4. Discussion

To our knowledge, this is the first Canadian study to show significant variations in how PHNs are treated across Canadian provinces. Our findings are consistent with observations made by other investigators who described significant variations in treatment modalities for patients with other solid tumors and with different sociodemographic characteristics [42]. In Canada, the number of transplants, resections, and ablations performed has roughly equaled the expected number of procedures for each year between 2002 and 2013. In contrast, the amount of transarterial embolization or chemoembolization performed has been consistently less than expected from 2005 onwards. Differences in treatment modalities might be due to many factors, including patients' comorbidities, tumor stage, patients' socioeconomic characteristics, local expertise, patients' preferences, and access to health care services [43].

Management of PHN is not only complex but also expensive, and most Canadian patients are referred to tertiary hospitals where multidisciplinary teams and advanced equipment are more often available than in community hospitals. However, the majority of Canadian hospitals are publicly funded with global budgets, and they do not receive any financial incentive for their clinical activity or efficiency [44]. Subsequently, complex medical and surgical conditions that require major health care resources become expenses rather than revenue generators like in other health care models such as in the United States [45]. Since the money does not follow patients, health care spending for the treatment of patients with hepatic tumors continues to raise financial concerns in institutions where operating budgets are fixed. Although the Canada Health Act (CHA) outlines the principle where medically necessary services should be equally guaranteed to every citizen independent of their socioeconomic status and location of their residence, financing of the Canadian health care system is accomplished not through a national system but provincial income taxes [46]. Because of this arrangement, Canadian provinces or territories with stronger economies are able to offer more health care resources than jurisdictions with less robust finances.

Because of the heterogeneity of resources available in different parts of Canada, we hypothesized that patients might receive different treatments for PHNs according to the geographical area where health care is delivered. Our hypothesis was confirmed by the findings of this study. In Atlantic Canada where provincial economies have underperformed for several decades [47], we found that most of the treatment modalities for PHNs had lower SIRs than in other regions. On the other hand, in Ontario, one of the most prosperous provinces, liver transplantation was performed 50% more frequently than the estimated national baseline and 134% more frequently than in British Columbia. There are several possible reasons for these findings. The first is that Ontario has two of the most active transplant programs in the country; one of those is among the largest programs for liver transplantation in the world. Therefore, it is conceivable that the specialists caring for patients PHNs in this region favored liver transplantation over other modalities since it is associated with the best five-year overall survival among all the other potentially curative interventions [48]. Other explanations include the fact that Ontario has one of the highest donation rates in Canada and many Canadians in need of a liver transplant from other provinces are often referred to one of the two transplant centers [49].

On the contrary, we found that SIR for liver transplantation in British Columbia was significantly lower than the national level despite the fact that per capita economic indices were comparable to Ontario and the Prairies [47]. One of the possible reasons for this finding was the relative low donation rate recorded during the study period in British Columbia, which ranged from 6 per million in 2005 to 14 per million in 2014 [49]. In comparison, donation rates during the same period in Ontario were 12 and 18 per million [49]. Because of the limited number of available grafts, patients with PHNs living in British Columbia might have been treated preferentially with hepatic resection or ablation techniques.

Overall, SIR for liver transplantation, hepatic resection, and ablation for primary hepatic tumors in Canada did not change during the study period. However, this was not true for transarterial locoregional therapies, for which SIR dropped from 4.4 (95% CI 3.3–5.5) in 2002 to 0.5 (95% CI 0.2–0.9) in 2005 and 0.2 (95% CI 0.1–0.4) in 2013.

This study had several limitations. First, data were provided by the national cancer registry and subject to collecting and reporting and classification errors [50]. This issue might have been more pronounced, as patients with PHNs are often diagnosed without histological confirmation by the combination of abnormally elevated serum tumor markers and characteristic features on cross-sectional contrast-enhanced imaging studies [13]. Therefore, a relatively large number of Canadians with PHNs might have not been biopsied and their diagnoses might have not been accurately registered in the national database. Second, data from Quebec were not available and therefore we were not able to provide a complete assessment of how PHNs are managed in Canada. Quebec is the second most populous province with 8,2 million inhabitants; consequently, our analysis did not include 23% of the Canadian population [38]. More importantly, record level data were not available, such that we were unable to correlate overall survival with each intervention and could not test the hypothesis that regional practice patterns resulted in different oncological outcomes. Also, the national cancer registry did not provide patients' sociodemographic characteristics nor the tumor staging at the time of diagnosis. Therefore, we were not able to assess if there were significant differences in patients' or tumors' characteristics at the time of diagnosis among Canadian provinces.

Nevertheless, several important considerations can be drawn from our study. Although health care in Canada is theoretically designed to provide similar treatment options for all patients, therapeutic interventions varied across different geographical areas. Disparities in health care resources and organ donation rates might be responsible for the heterogeneity in the treatments delivered to Canadians with PHN. To our knowledge, this is the first study to demonstrate such marked geographic differences in the treatment of PHN in Canada. The underlying causes of these observations were beyond the scope of this study, as health care decisions influencing the management of patients with PHNs are complex and depend on patient characteristics, tumor stage, underlying liver disease, local expertise, patient preferences, and other factors, including health care resources and organ donation rates [51, 52].

In conclusion, our analysis demonstrated that in Canada there are geographic-specific differences in treatment of PHN. Future studies should focus on determining the underlying causes for these disparities and whether there are differences in oncological outcomes.

## Figures and Tables

**Table 1:** Summary of codes used to identify treatment modalities of Canadian patients with primary hepatic cancer during the period between 2002 and 2013. Codes for liver transplantation, hepatic resection (segmental or subtotal), ablation, and embolization with or without chemotherapy or transarterial radiation were selected from the Canadian Classification of Health Interventions manual [38].

Treatment modality	Code
*Liver transplantation* (cadaveric or live donor graft, whole organ or split)	1.OA.85.^∧∧^
*Hepatectomy* (laparoscopic or open surgery, segmental or subtotal)	1.OA.87.^∧∧^
*Ablation* (percutaneous, laparoscopic, open surgery, acetic acid, alcohol, cryoablation, radiofrequency ablation, microwave ablation)	1.OA.59.^∧∧^
*Chemotherapy* (transarterial chemotherapy)	1.0A.35^∧∧^
*Embolization* (transarterial with or without chemotherapy or radiation therapy)	1.OA.13^∧∧^
